# Systems biology approaches in the study of Chinese herbal formulae

**DOI:** 10.1186/s13020-018-0221-x

**Published:** 2018-12-29

**Authors:** Fei-Fei Cai, Wen-Jun Zhou, Rong Wu, Shi-Bing Su

**Affiliations:** Research Center for Traditional Chinese Medicine Complexity System, Shanghai University of Traditional Chinese Medicine, Shanghai, 201203 China

**Keywords:** Systems biology, Chinese herbal formulae, Chinese medicine, Omics

## Abstract

Systems biology is an academic field that attempts to integrate different levels of information to understand how biological systems function. It is the study of the composition of all components of a biological system and their interactions under specific conditions. The core of systems biology is holistic and systematic research, which is different from the manner of thinking and research of all other branches of biology to date. Chinese herbal formulae (CHF) are the main form of Chinese medicine and are composed of single Chinese herbal medicines (CHMs) with pharmacological and pharmacodynamic compatibility. When single CHMs are combined into CHF, the result is different from the original effect of a single drug and can be better adapted to more diseases with complex symptoms. CHF represent a complex system with multiple components, targets and effects. Therefore, the use of systems biology is conducive to revealing the complex characteristics of CHF. With the rapid development of omics technologies, systems biology has been widely and increasingly applied to the study of the basis of the pharmacological substances, action targets and mechanisms of CHF. To meet the challenges of multiomics synthesis-intensive studies and system dynamics research in CHF, this paper reviews the common techniques of genomics, transcriptomics, proteomics, metabolomics, and metagenomics and their applications in research on CHF.

## Introduction

Chinese herbal formulae (CHF) are the main forms of prescription for the clinical application of Chinese medicine (CM) and embody the holistic philosophy of CM and the characteristics of treatment according to syndrome differentiation. The complexity of the chemical compositions and the diversity of the prescription compatibility and therapeutic functions of CHF have long presented great difficulties in the research of CM [1]. In contrast to the single compounds of Western medicine, which have specific targets and definite modes of action, CHF contain many types of components. The effects of these chemical components not only are superimposed but also interact with each other through multiple targets, pathways and mechanisms [2, 3]. Slow progress in the research on the pharmacological mechanisms of CHF has hindered their application and popularization throughout the world, thus becoming one of the key scientific problems to be solved in the modernization of CM.

**Fig. 1:**
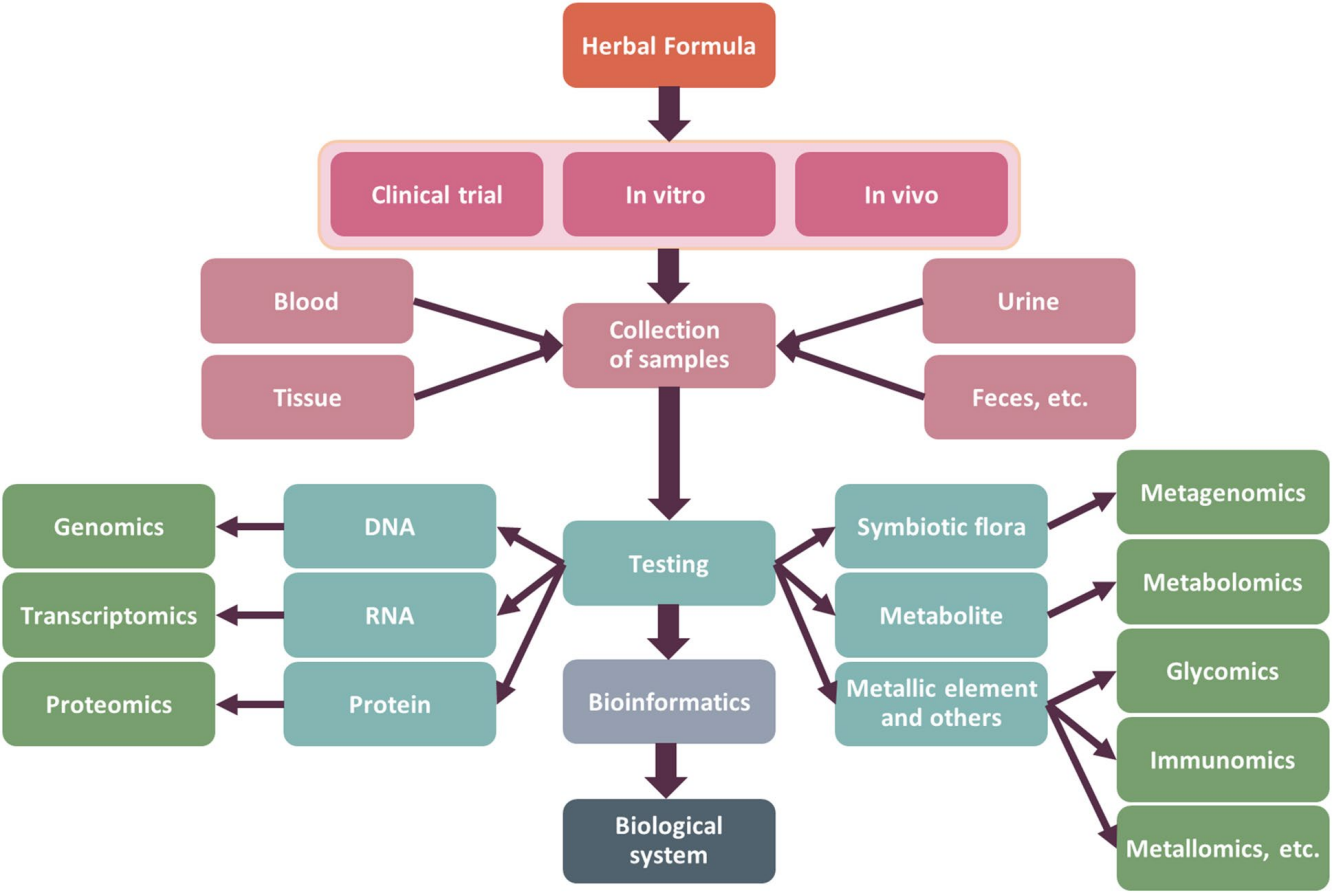
Overview of the applications of systems biology in CHF research

Systems biology studies the interactions among different parts of a biological system at the cellular, tissue, organ and biological levels, and it quantitatively describes and predicts biological functions, phenotypes and behaviors through bioinformatics and through the computational and mathematical modeling of complex biological systems [4, 5]. Systems biology is an interdisciplinary field of study that focuses on complex interactions within biological systems, using a holistic approach instead of the more traditional reductionism of biological research. Systems biology is characterized by holistic and systematic research and explores the laws of life “from surface to point”, corresponding to the holistic view of CM. The process of applying systems biology for CHF research is summarized and illustrated in Fig. 1.

With the development of high-throughput technologies and data analysis methods, such as genomics, proteomics, metabolomics and transcriptomics, an increasing number of studies have focused on clarifying complex biological phenomena at the systems level [6, 7]. Large amounts of information from omics technologies and computational studies, including bioinformatics, data mining and machine learning, have been used to understand biological phenomena to predict interactions of biological systems [8, 9]. As a complement to the traditional research mode, a novel CM pharmacological approach through the combination of network pharmacology, omics technologies and computational studies has been established to update the research paradigm from the current “one target, one drug” mode to a new “network target, multicomponents” mode [10]. The approach can discover the combinatorial rules and network regulation effects of CHF from a systems perspective and at the molecular level by prioritizing disease-associated genes, predicting the target profiles and pharmacological actions of herbal compounds, revealing drug-gene-disease comodule associations, screening synergistic multicompounds from CHF in a high-throughput manner [11], analyzing data through computational studies [12], and then clarifying the combinatorial rules and network regulation effects of CHF. Therefore, this article focuses on an overview of systems biology technology and the application of systems biology in CHF studies.

## Application of genomics in CHF research

Genomics is an interdisciplinary field of science focusing on genome mapping, nucleotide sequencing and gene function analysis [13]. According to the time of development and research purposes, genomics is divided into the four branches of structural genomics, functional genomics, comparative genomics and pharmacogenomics.

### Common techniques in genomics

Commonly used research methods in genomics include DNA sequencing and bioarray technology. According to different sequencing objects, DNA sequencing technology can be divided into the following categories. (1) Genome-wide association study (GWAS) typically focuses on associations between single-nucleotide polymorphisms (SNPs) or copy-number variations (CNVs) and traits [14]. (2) Exome sequencing uses sequence capture technology to capture and enrich genome-wide exon-region DNA for high-throughput sequencing [15], which can be used to find pathogenic and susceptible genes of complex diseases. (3) Methylated DNA immunoprecipitation sequencing (MeDIP-Seq) can quickly and effectively find the methylation regions in the genome, thus enabling a comparison of the differences in DNA methylation modification patterns between samples [16]. (4) Chromatin immunoprecipitation (ChIP) is used to investigate the interaction between proteins and DNA within cells and to determine the specific location in the genome with which various histone modifications are associated, indicating the targets of histone modifiers [17]. Genomics involves chip technology, including the single-nucleotide polymorphism array (SNP-A), array-based comparative genomic hybridization chip (aCGH), and methylated DNA immunoprecipitation chip (MeDIP-Chip). In addition to high-throughput sequencing and microarray chip technology, low-throughput technology, such as real-time fluorescence quantitative polymerase chain reaction (RT-PCR) and the TaqMan probe, are also needed for verification of genomics research.

### Application

Genomics technology has the characteristics of being high-throughput, multifactorial, rapid and sensitive [18], and it can be used to systematically study the multicomponent, multipathway and multitarget effects of CHF systematically.

Current studies have reported that genomics is used to evaluate the efficacy of CHF and to reveal the mechanism of action of CHF at multiple levels. Wen et al. [19] used microarray technology to compare the differences in gene expression between the Siwu decoction and estradiol in the human breast cancer cell line MCF-7, using a microarray and a connectivity map to evaluate the efficacy of the Siwu decoction and explore its molecular mechanism of action. The authors found that the Nrf2-mediated oxidative stress pathway might explain the mechanism of Siwu decoction’s cancer defense by using IPA software, and RT-PCR confirmed the overexpression of five genes in this pathway. This study demonstrates the feasibility of microarray technology in combination with other techniques for the evaluation of therapeutic efficiencies and mechanism studies of CHF. Wang et al. [20] reported the mechanism of the Danqi pill (DQP) in myocardial ischemia treatment by measuring gene microarrays in a rat model. After evaluating the effects of signaling pathways and metabolism, the authors found that DQP can reverse energy metabolic disorders to recover cardiac function.

In addition, genomics has been used to analyze therapeutic targets of CHF and to predict other potential drugs. Tang et al. [21] examined therapy using Sini decoction (SND) on kidney yang deficiency in a rat model. Using an mRNA microarray with enrichment analysis, Wang et al. found that SND treatment can improve hypothalamic–pituitary–adrenal axis hormones. Kim et al. [22] connected GWAS diseases to CM by analyzing microarray gene expression and constructing disease-gene networks. The authors analyzed 14,713 GWAS disease-CM-target gene pairs to propose potential drugs from CM. Considering that molecular docking technology may lead to omitted or incorrect information, Zhang et al. [23] proposed the GEPSI method to identify the target proteins of CM by calculating the similarities of gene expression.

These reports are promising for the application of DNA microarray technology in the research of CM. However, other high-throughput technologies such as exome sequencing, MeDIP-Seq, ChIP, aCGH and SNP-A have not been used extensively to date in the study of CHF and, hence, need further research. It is expected that genomics or genomics combined with other omics analyses will offer comparatively high practical value with a systematic flow in the study of CHF.

## Application of transcriptomics in CHF research

Transcriptomics technologies are techniques used to study gene transcription and transcriptional regulation. Unlike the static genome, the transcriptome is regulated by both exogenous and endogenous factors. Therefore, the transcriptome is a dynamic link between the genome of a species and its external physical characteristics, reflecting the expression level of all genes in a particular organ, tissue or cell at a particular physiological stage. The current methods for obtaining and analyzing transcriptome data can be divided into two categories: targeted and untargeted methods [24].

### Targeted methods

Targeted methods are used to analyze the expression of individual RNA sequences, including gene expression profiling chips, mi-RNA microarrays, long noncoding RNA microarrays (LncRNA Microarray), cDNA-amplified fragment length polymorphism (cDNA-AFLP), Northern blotting and in situ hybridization. The advantages of cDNA-AFLP technology are its repeatability, low false-positive rate and ability to detect the expression of mRNA in low abundance, while the associated difficulty is the selection of suitable endonuclease combinations [25]. Northern blotting is mainly applied to studies of the dynamic expression of specific trait genes at the mRNA level. Using in situ hybridization technology to detect the expression of miRNA can provide a more intuitive display of the spatial and temporal expression patterns of miRNA.

Cui et al. [26] identified differentially expressed genes in Buyang Huanwu decoction intracerebral hemorrhage treatment with lncRNA and mRNA microarray technology and verified the results by qRT-PCR. The biological functions and signal transduction pathways of the differentially expressed genes were analyzed and were found to be related to hemoglobin complexes, oxygen transporters, oxygen transporters, and pyruvate metabolism. Wang et al. [27] confirmed that the Jian-Pi-Zhi-Dong decoction could effectively inhibit the abnormal behavior of mice with Tourette syndrome and increase the levels of dopamine transporter proteins and mRNA in striatum by immunohistochemistry and in situ hybridization. Dai et al. [28] identified microRNAs-223-3p as the key microRNAs in Qi-Shen-Yi-Qi dripping pills that regulate the angiogenesis of ischemic myocardial microvascular endothelial cells by miRNA chip and real-time PCR techniques.

### Untargeted methods

Untargeted methods analyze the expression of a large number of different RNA sequences and perform extensive screening, including expressed sequence tags (EST), serial analysis of gene expression (SAGE), massively parallel signature sequencing (MPSS), and RNA sequencing (RNA-seq). EST can analyze a large sample number and can be used for cross-species comparative analysis; however, the data volume is large, and the error rate is high. SAGE is not only qualitative but also quantitative for gene differences, but one significant drawback of this technique is that it requires a large amount of mRNA. MPSS is simple and efficient and can provide information on terminal sequences, and it is suitable for any organism; however, the detection cost is high. RNA-seq with digital signaling has high sensitivity and quantitative accuracy.

The transcriptome is characterized by time specificity, tissue specificity and spatial specificity. In a transcriptional analysis of changes in *Candida albicans* gene expression due to treatment with a Huanglian Jiedu decoction (HLJDD) performed by an RNA-seq technique, Yang et al. [29] found that 735 differentially expressed genes were identified through gene expression analysis, including 700 upregulated genes and 35 downregulated genes. Through the functional annotation analysis of differentially expressed genes, 26 important pathways for HLJDD inhibition of *Candida albicans* infection were identified, especially those occurring through DNA replication and transporter activity pathways. However, to enable the transcriptome to play a greater role in the study of CHF, more attention should be paid to the study of gene modification and protein modification to deepen the understanding of the function and structure of functional genes and regulatory genes.

## Application of proteomics in CHF research

The term “proteome” was coined in 1994 and is defined as the protein complement of a genome [30]. Proteomics is an extension of the concept of the proteome and generally refers to the large-scale study of proteins and proteomes, including protein expression levels, post-transcriptional modifications and interactions [31].

### Separation and detection techniques in proteomics

The separation techniques in proteomics research include two-dimensional gel electrophoresis (2DGE), fluorescence two-dimensional differential gel electrophoresis (2-D DIGE), multidimensional liquid chromatography (MDLC), and capillary electrophoresis (CE). Among these techniques, 2DGE is the most widely used separation technology in proteomics research. The technique can solve the problem of repeatability and reduce the human error associated with gel contrast analysis. 2DGE is suitable for comparing the differences in protein expression between two samples with high sensitivity, but it is expensive [32]. MDLC can be directly linked to mass spectrometry (MS), easily realizing automation and high throughput and avoiding limitations associated with the molecular weight and isoelectric point. However, MDLC is not as intuitive as gel electrophoresis for visualizing protein spots, and its resolution and reproducibility are not as good as those of 2DGE. CE combines electrophoretic separation with chromatographic separation technology. The technique has advantages in sensitivity, separation efficiency and cost, but it is difficult to analyze proteins with high molecular weights using this approach.

Proteomics detection methods include MS, stable isotope labeling, tandem affinity purification (TAP), yeast two-hybrid assays (YTH), protein chips, X-ray crystallography (XRC), and nuclear magnetic resonance (NMR). Among these methods, MS includes matrix-assisted laser desorption ionization (MALDI), surface-enhanced laser desorption ionization (SELDI), and electrospray ionization (ESI). MALDI is suitable for detecting the molecular mass of peptide segments but not peptide sequences. ESI–MS has a shorter detection time and higher sensitivity and resolution than MALDI, but it requires higher sample purity and more complex data acquisition and analysis. SELDI-TOF–MS is a surface-enhanced MS based on MALDI and is an analytical chip used for expression spectrum analysis. Stable isotope labeling includes stable isotope labeling with amino acids in cell culture (SILAC), isotope-coded affinity tags (ICAT), and isobaric tags for relative and absolute quantification (iTRAQ). The limitation of SILAC is that it can only be used for cells. ICAT can only detect cysteine-containing proteins, while iTRAQ can only achieve relative quantification.

### Application

According to the occurrence and development of diseases, CHF mostly function at the protein level [33]. Proteomics research overcomes the nonlinear relationship between protein expression and genes and studies the mechanism and target of CHF action directly at the protein level. Proteomics technology, as the main method of large-scale research on proteins, is applied mainly in two respects: to protein expression profile differences and to protein structure, function and interaction analysis.

#### Analysis of protein profiles in CHF research

Many studies use proteomics to study the changes in protein spectrum before and after intervention of CHF and to evaluate the pharmacodynamics of CHF at the overall level. A study of the Dingxin recipe in rats with ischemia/reperfusion-induced arrhythmias was carried out by Jia et al. [34]. The authors identified differentially expressed proteins using 2DGE and MALDI-TOF–MS and then validated those proteins by immunohistochemistry, qRT-PCR, western blotting and enzyme-linked immunosorbent assays, indicating that the effect of the Dingxin recipe on arrhythmia induced by ischemia/reperfusion may be related to the increase in prohibitin expression inhibiting neutrophil infiltration and IL-6 expression. Fan et al. [35] determined the protein expression profiles of rat mesenchymal stem cells and cardiomyocyte-like cells by the 2DGE technique and found that the Shuanglong formula could induce mesenchymal stem cells to transform into cardiomyocyte-like cells. In the same vein, the determination of protein expression profiles was conducted in research on the effect of the Fuzheng Huayu formula [36] and Yinchenhao decoction [37] on the proteome of fibrotic livers, as well as the effects of the ZiBu PiYin recipe [38] and Tianqi Jiangtang capsule on diabetes, of Yuanshi Shengmai Chenggu tablets on avascular osteonecrosis of the femoral head [39], of Jie-Geng-Tang on lipopolysaccharide-induced acute lung injury in mice [40], and of the Tao Hong Si Wu decoction against ischemia reperfusion injury [41].

#### Functional analysis of proteins in CHF research

Protein changes in the body may be the result or the cause of disease; therefore, abnormal proteins and their interactions during the disease course may be potential targets for CHF. In research carried out by Tang et al. [42], protein–protein interaction (PPI) networks containing MMP-9 protein data were obtained from proteomic data published in a database and were further analyzed by high-throughput virtual screening, identifying three compounds that bind to the zinc-binding site of MMP-9 with predictable activity. The researchers then proposed three CHFs containing these compounds for increasing the activity of MMP-9 proteins and thus reducing the side effects of tetracycline. Liu et al. [43] coupled iTRAQ with 2-D LC–MS/MS to identify the differentially expressed proteins in serum between Zhibai Dihuang granule-treated rats and yin-deficiency heat syndrome rats and analyzed the differential protein functions via bioinformatics, finding that Zhibai Dihuang granules may alleviate yin-deficiency heat syndrome by regulating complementary activation and inflammation, enhancing the body’s ability to recognize antigens.

## Application of metabolomics in CHF research

Metabolomics, which emerged in the 1990s, studies the metabolic regulatory networks of organisms by examining their metabolic products and dynamic changes in the course of diseases. Metabolomics is widely used in the study of pathophysiological changes of diseases. By evaluating the differential expression of various endogenous substances, such as blood and urine, much information about disease diagnosis and drug efficacy can be provided [44]. In metabolomics studies, the analytical techniques for samples are mainly ^1^H-NMR and MS.

### NMR

NMR enables noninvasive, unbiased detection of samples and is responsive to hydrogen-containing compounds; thus, the technique can detect as many compounds as possible in a sample. However, the sensitivity of NMR is lower than that of mass spectrometry. Wei et al. [45] studied the therapeutic effect of the HLJDD decoction on acute pancreatitis by comparing two models of cholestasis injury induced by bile duct ligation and thioacetamide. NMR-based metabolomic and pathological studies showed that *Coptis chinensis* and its main alkaloid, berberine, could inhibit inflammatory factors and protect the liver. Combining these methods with ^1^H-NMR and network analysis, Zhang et al. successfully revealed that the three main compounds in HLJDD, berberine, baicalin and jasmine, can improve metabolic disorders of ischemic stroke by improving metabolic abnormalities and regulating oxidative stress, neuronal autophagy and inflammatory responses [46]. The therapeutic effect of HLJDD and its four variants on a septic cecum ligation and perforation (CLP) model was studied by ^1^H-NMR, histological examination, biochemical examination and molecular biology. The results showed that HLJDD had a better therapeutic effect in the CLP model than its four variants and that the HMGB-1/TLR4/NF-kappa B signaling pathway may be involved in HLJDD’s ability to reduce tissue damage and improve metabolic disorders in septic rats [47].

### GS–MS

It is difficult to simultaneously determine the metabolites coexisting in biological systems at very different concentrations. Commonly used separation techniques include gas chromatography (GC), liquid chromatography (LC) and CE. Analytes in metabolomics samples contain highly complex mixtures. By separating some analytes from others, complex mixtures can be simplified before testing. The separation procedure is not mandatory and is usually omitted in NMR. Because existing analytical techniques each have their own advantages and scope of application, the strategy for metabolomics comprehensive analysis is to combine separation and analytical techniques. GC–MS is suitable for the analysis of metabolites with low molecular weights, low polarity and low boiling points or volatile substances after derivatization.

GC–MS is a feasible way to systematically study the therapeutic effect of CHF. The Xuefu Zhuyu Decoction (XFZY) has a distinct therapeutic effect on traumatic brain injury (TBI) in rats. Feng et al. used GC–MS to analyze the plasma metabolomics of sham, vehicle and XFZY groups by univariate and multivariate statistical analysis to illustrate the therapeutic approach of XFZY in TBI. The authors concluded that XFZY treatment can alleviate neurological impairment and cortical lesion volume on the third day after brain injury and reverse the abnormalities of plasma metabolites such as glutamate, lactic acid, 3-hydroxybutyric acid and ribitol [48]. GC-TOF–MS was used to evaluate the efficacy and mechanism of the Shenfu decoction in the treatment of chronic heart failure induced by coronary artery ligation in rats. Unsupervised principal component analysis showed that CHF significantly altered the fingerprint of urinary metabolites. After SFD treatment, the metabolomic profiles of CHF rats were significantly reversed, and the pathways of fatty acid biosynthesis, fatty acid elongation, steroid biosynthesis, galactose metabolism and amino acid metabolism in rats were significantly altered [49].

### LC–MS

LC–MS is suitable for the analysis of high-molecular-weight, thermally instable and high-boiling-point compounds. Based on LC-TOF/MS and LC-QqQ/MS, a nontarget metabolomics-driven method for the rapid screening and identification of xenobiotics and related metabolites in vitro was developed. Using this method, Wu et al. [50] found that iridoid glycosides, monoterpenoids, flavonoid glycosides, and anthraquinones are the main absorbed chemical components of the Zhi-Zi-Da-Huang decoction and that hydrolysis, glucuronidation and sulfation are the main metabolic pathways in vivo. Yan et al. [51] applied UHPLC-LTQ-Orbitrap MS combined with a spike-in method to the study of Danqi Tongmai tablets for the preconditioning of acute myocardial ischemia (AMI) rats, which indicated that pretreatment with this CHF can partially regulate disordered TCA circulation and amino acid and nucleotide metabolism, thereby reducing injury from AMI. In addition, the effect and mechanism of Jinxin oral liquid in treating viral pneumonia caused by the respiratory syncytial virus via improving lipid metabolism disorders [52], the protective effect of low-dose Sini decoction against myocardial injury induced by isoproterenol [53], the inhibitory effect of the Yinchenhao decoction toward dimethylnitrosamine-induced liver fibrosis in rats [54], and the improvement from the modified Jiu Wei Qiang Huo decoction on H1N1-virus pneumonia in mice [55] have all been well verified by LC–MS technology.

Regardless of which analytical technology is adopted, no single technique can completely cover all metabolomics compounds [56]. In recent years, researchers have tried to integrate various analytical techniques to give full play to the advantages of various methods. LC–MS and GC–MS analyses were performed to quantitatively evaluate the compatibility of CHF from the perspective of overall metabolic profiles and specific metabolites [57, 58]. These studies suggest that appropriate analytical techniques need to be selected before metabolomics studies on CHF can be carried out so that the results can be more comprehensive and accurate. Metabolites are not confined to the substrates and products of certain enzymes in the metabolic pathway. Instead, metabolites act as structural units, signaling molecules, and regulatory factors and play many other roles and interact in the form of a metabolic network in life activities.

## Application of metagenomics in CHF research

Metagenomics is a microbial research method based on the genome of microbial populations in environmental samples, aiming at examining microbial diversity, population structure, evolutionary relationships, functional activities, interactions and environmental relationships. Functional gene screening and sequencing analysis are the research means.

### Common techniques in metagenomics

At present, the main application of metagenomics in-clinic is based on gene sequence analysis of microbial species and compositions. 16S rRNA gene cloning and sequencing, PCR-denaturing gradient gel electrophoresis (PCR-DGGE), PCR-temperature gradient gel electrophoresis (PCR-TGGE), terminal restriction fragment length polymorphism (T-RFLP), and next-generation sequencing technology such as 454 and Illumina have been widely used in metagenomics research. High-throughput, large-scale, in-depth sequencing combined with multivariate statistical methods can provide more direct information on the composition and function of microorganisms and identify specific bacterial groups closely related to the physiological and pathological state of the organism. 16S rRNA gene cloning and sequencing can not only analyze the species of bacteria in a sample but can also reflect the proportion of various bacteria and provide relative quantitative analysis [59]. PCR-DGGE has a high resolution and can detect mutant individuals with single-base differences. However, when the length of DNA fragments detected by PCR-DGGE exceeds 500 bp, the resolution will decrease [60]. Similarly to DGGE, TGGE can only analyze DNA fragments shorter than 500 base pairs, and there are only ten to twenty bands in the map reflecting the dominant flora in the community, while the disadvantaged flora cannot be detected. T-RFLP was established based on PCR but without the isolation and cultivation of bacteria. However, when two distinct sequences share a terminal restriction site, they cannot be distinguished and only show one peak on an electropherogram.

### Expectations of the application of metagenomics in CHF

One of the main methods for the application of metagenomics in CM research is to construct a 16S rRNA gene library for flora analysis based on the species specificity of the 16S rRNA gene. Another common method is to extract the total nucleic acid from bacteria and amplify the 16S rRNA gene by PCR, then analyze the data by molecular biology techniques such as fingerprinting combined with multivariate statistical analysis. To study the effect of Xiexin Tang on the distribution of intestinal flora in rats with type 2 diabetes mellitus (T2DM), Wei et al. [61] utilized high-throughput 16S rRNA gene sequencing to detect the cecum samples of the rats. Sequencing analysis showed that certain short-chain fatty acid-producing and anti-inflammatory bacteria in the intestinal microflora of T2DM rats were significantly altered after Xiexin Tang intervention, and these microbiotas were closely related to the changes in related indices of T2DM. Tong et al. [62] proved the effect of metformin and a specially designed CHF on T2DM with hyperlipidemia through a randomized clinical trial. Then, Illumina sequencing and multivariate statistical methods were used to analyze the V3 and V4 regions of 16S rRNA genes to evaluate changes in the intestinal microbial structure. The results suggest that the combination of metformin and CHF may improve T2DM with hyperlipidemia by enriching Brucella and fecal bacilli and other beneficial bacteria. To verify that oil tea can induce changes in intestinal microbes and play an anti-diabetic role, Lin et al. [63] carried out 16S rRNA gene sequencing on fecal samples of db/db mice fed oral salt, metformin and oil tea. The results showed that Lachnospiraceae were significantly enriched after oil tea treatment and correlated with decrease indicators related to diabetes. Gao et al. [64] studied the effects of S-3-1, a homogeneous polysaccharide purified from the Sijunzi decoction, on human intestinal microflora and short-chain fatty acids by a GC technique, analyzing the V3 and V4 regions of 16S rRNA after Illumina MiSeq sequencing. It appeared that S-3-1 could regulate the abundance of 9 intestinal flora, while S-3-1 incubated in gastric and intestinal juice enhanced the ability to regulate the composition of intestinal flora and regulated 13 types of bacteria genera to play an immunoregulatory role.

The number of microorganisms in the human body is more than ten times that of body cells, and the corresponding genetic code is 100 times the size of the human genome [65]. Most existing studies have focused on the distribution of intestinal microorganisms. We should pay more attention to the impact of intestinal microecological changes on human health and disease; that is, we should pay more attention to the functions of microorganisms. There is a great challenge and opportunity in the deeper and wider application of metagenomics in CHF research to connect the functional changes of intestinal flora with the influence of CHF on microflora.

## Comprehensive application of multiple omics methods in CHF research

Considering that the combination of multiple omics methods will enable the comprehensive evaluation of the efficacy and complex mechanisms of CHF, Du et al. [66] used transcriptomics, metabolomics, and pharmacodynamics to observe the therapeutic effect of the Baoyuan decoction on myocardial infarction in rats with left anterior descending coronary artery ligation and investigate its molecular mechanism. Their work successfully revealed the mechanism of multiple pathways regulating the cardioprotective effects of the Baoyuan decoction. Huang et al. [67] established a cold-stagnation and blood-stasis primary dysmenorrhea rat model to investigate the effect and mechanism of the Shaofu Zhuyu decoction. In their work, metabolic profiling was analyzed by LC–MS, and the correlation between biomarkers and biochemical indicators was also analyzed. Meanwhile, peripheral blood mononuclear cells were isolated, and their transcript levels were quantified by RT-PCR. The findings suggested that SFZYD regulated the MAPK pathway and thus improved the metabolic profiles and biochemical parameters in cold-stagnation and blood-stasis primary dysmenorrhea rats. Sun et al. [68] carried out a multilevel evaluation of the Qishe pill by metabolomics, genomics and proteomics. According to their constitutional types, 108 subjects were divided into qi-deficiency, yin-deficiency and blood-stasis groups. ^1^H-NMR, UHPLC-MS, the Human-CoreExome + v1.1-Psych Array, and Illumina’s HT-12 bead chips were used to establish and verify a population pharmacokinetic (PopPK) model for the Qishe pill in the three groups, providing personalized medicine strategies for the application of CHF. Zhao et al. [69] identified a group of short-chain fatty acid-producing bacteria at the strain level that can increase insulin secretion and enhance insulin sensitivity by combining metagenomics analysis with metabolic levels in patients with T2DM. These bacteria can be regarded as a necessary “ecological functional group” for restoring and maintaining human health. The team found that the higher the restored abundance and diversity of these bacteria were, the lower the reduction of HbA1c became, and they established a statistical model to predict the efficacy of these key early bacterial changes.

## Perspectives

Clinically effective CHF usually exerts therapeutic effects by regulating multiple targets and affecting multiple pathways. At present, the evaluation system for the clinical efficacy of CHF still needs to be improved. It is difficult to explain the efficacy of CHF systematically and comprehensively by traditional evaluation methods that only use a single index or a few indices. The evaluation of CHF efficacy combined with the integrity and dynamics of systems biology should still be based on CM syndrome differentiation and treatment concepts. In studies of the mechanisms of CHF, in addition to reflecting the pharmacodynamic substance basis of CHF from the aspects of genes, proteins, and metabolites, the bioinformatics method should be used to integrate and construct a biological network map of the mechanism of the CHF therapeutic effects and to clarify the mechanism of CHF therapeutic effects from the perspective of the organism as a whole to replace the study of pharmacodynamic mechanisms from only the molecular perspective. Compared with traditional research techniques, the acquisition and analysis of high-throughput, large-scale data makes the research of systems biology better aligned with the characteristics of CHF. Determining how to carry out system dynamics research of CHF and combine traditional pharmacodynamic and pharmacokinetic methods to assess CHF efficacies and mechanisms remains a big challenge for future research and applications.

Although each omics method exists independently, it reflects biological characteristics from different angles. In specific studies, we should select methods and techniques according to the research objects and purposes. The study of CHF by a single omics method is usually not sufficiently comprehensive. Findings should be mutually validated in combination with multiple -omics methods, and even multicenter, multilevel research strategies should be used to identify changes in gene-protein-metabolite level consistency. Although individualized precision medicine still has a long way to go, CHF treatment based on the molecular subtyping of CM syndrome differentiation as well as holistic evaluation based on systematic pharmacology/network pharmacology are the existing research and application approaches. With the powerful tool of systems biology, CM can rapidly promote the development of personalized medicine.

## Abbreviations

CHF: Chinese herbal formulae
CM: Chinese medicine
CHM: Chinese herbal medicine
GWAS: genome-wide association study
SNPs: single-nucleotide polymorphisms
CNVs: copy-number variations
MeDIP-Seq: methylated DNA immunoprecipitation sequencing
ChIP: chromatin immunoprecipitation
aCGH: array-based comparative genomic hybridization chip
MeDIP-Chip: methylated DNA immunoprecipitation chip
RT-PCR: real-time fluorescence quantitative polymerase chain reaction
LncRNA Microarray: long noncoding RNA microarray
cDNA-AFLP: cDNA-amplified fragment length polymorphism
EST: expressed sequence tags
SAGE: serial analysis of gene expression
MPSS: massively parallel signature sequencing
RNA-seq: RNA sequencing
2DGE: two-dimensional gel electrophoresis
CE: capillary electrophoresis
MS: mass spectrometry
XRC: X-ray crystallography
NMR: nuclear magnetic resonance
ESI: electrospray ionization
iTRAQ: isobaric tags for relative and absolute quantification
GC: gas chromatography
LC: liquid chromatography
